# The implementation gap in critical care: From nutrition to ventilation

**DOI:** 10.2478/jccm-2025-0011

**Published:** 2025-01-31

**Authors:** Razvan Azamfirei

**Affiliations:** Department of Anesthesiology and Critical Care, University of Pennsylvania Perelman School of Medicine, Philadelphia, USA

Critical care medicine pushes boundaries. We talk about personalized medicine and wax poetic on sophisticated trial design, all while debating using diaphragmatic ultrasound for ventilator weaning. Our excitement about the latest mechanical circulatory support device or novel vasopressor is matched only by the rush to share the latest “groundbreaking” meta-analysis – inevitably analyzing the same five trials as the last one, just with a different statistical twist. None of this is to say that such discussions do not have merit. But our fascination with tomorrow's breakthroughs disguises a more fundamental challenge: we consistently fail to deliver basic, routine care at the bedside.

ICU nutrition demonstrates this pattern of failure. Patients consistently receive less than 50% of prescribed calories and protein, with feeds frequently interrupted [1, 2]. When nutrition is initiated, patients rarely achieve nutritional targets within the first week of critical illness, a period when metabolic demands peak and nutritional adequacy matters most [2,3,4]. Only 3–5% of ICUs successfully achieve average adequacy of calories and protein for their patients [3]. One may argue that benchmarking ICU performance in nutrition delivery is unfair – after decades of research, we lack substantive evidence for nutritional interventions, optimal delivery routes, or even appropriate targets [5,6,7]. While we envision artificial intelligence-driven protocols and bedside genomics to personalize nutrition, we struggle to meet nutritional needs or maintain consistent feeding schedules [8]. It is indeed worrying that for such a fundamental component of critical care, we continue to engage in vibes-based medicine. But the reality remains: we neither know what proper nutrition looks like nor can we provide what little we do know.

This implementation gap extends beyond areas of scientific uncertainty to interventions with clear evidence. The ABCDEF bundle comes with strong recommendations from the Society of Critical Care Medicine and documented associations with decreased mortality and shorter lengths of mechanical ventilation [9, 10]. Yet ICUs consistently struggle with implementation: only 36% have fully implemented comprehensive pain protocols, and just 42% have fully operational delirium management protocols [11, 12]. Even in ICUs with established practices, actual delivery is disappointing – 43% of ICU days lack delirium screening [13]. One might say the ABCDEF bundle is a complex intervention and difficult to implement. True enough, but the struggle with implementation becomes even more striking when we consider mechanical ventilation.

For more than 25 years, since the landmark ARDSNet trials, we have known that low tidal volume ventilation reduces mortality in ARDS [14]. This finding has been demonstrated in successive trials, adapted to the general ICU population, and is accepted almost universally as best practice. The intervention requires no special equipment, just attention to basic ventilator settings, and has clear mortality benefits. Yet repeated studies find only 19.3–31.4% of ARDS patients receive low tidal volume ventilation [15,16,17]. This failure cannot be attributed to complex protocols or uncertain evidence – we know what to do, we just don't do it.

I use these examples as a reminder that for care to be effective, it must first be delivered. Futuristic critical care is appealing, and pursuing innovations isn't inherently problematic; our field can address more than one thing at once. But this pursuit of tomorrow's innovations is often a form of escapism from today's implementation failures. On our way to AI-driven care algorithms and bedside genomics, we should be mindful of the care our patients are not receiving.

Critical care advances through both discovery and delivery. Until we improve at our core task – delivering proven therapies consistently and effectively at the bedside – the promise of personalized medicine will remain just that: a promise. Our patients deserve better than waiting for tomorrow's innovations while we fail to deliver today's standard of care.
